# Supplementary material for: Recommendations to Enhance Parental Involvement and Adolescent Participation in Physical Activity
Source: Int J Environ Res Public Health. 2022 Jan 25;19(3):1333. doi: 10.3390/ijerph19031333 (PMC8835355; doi:10.3390/ijerph19031333)
Supplement: Supplementary file 1 [file ijerph-19-01333-s001.zip › ijerph-1462863-supplementary.pdf]

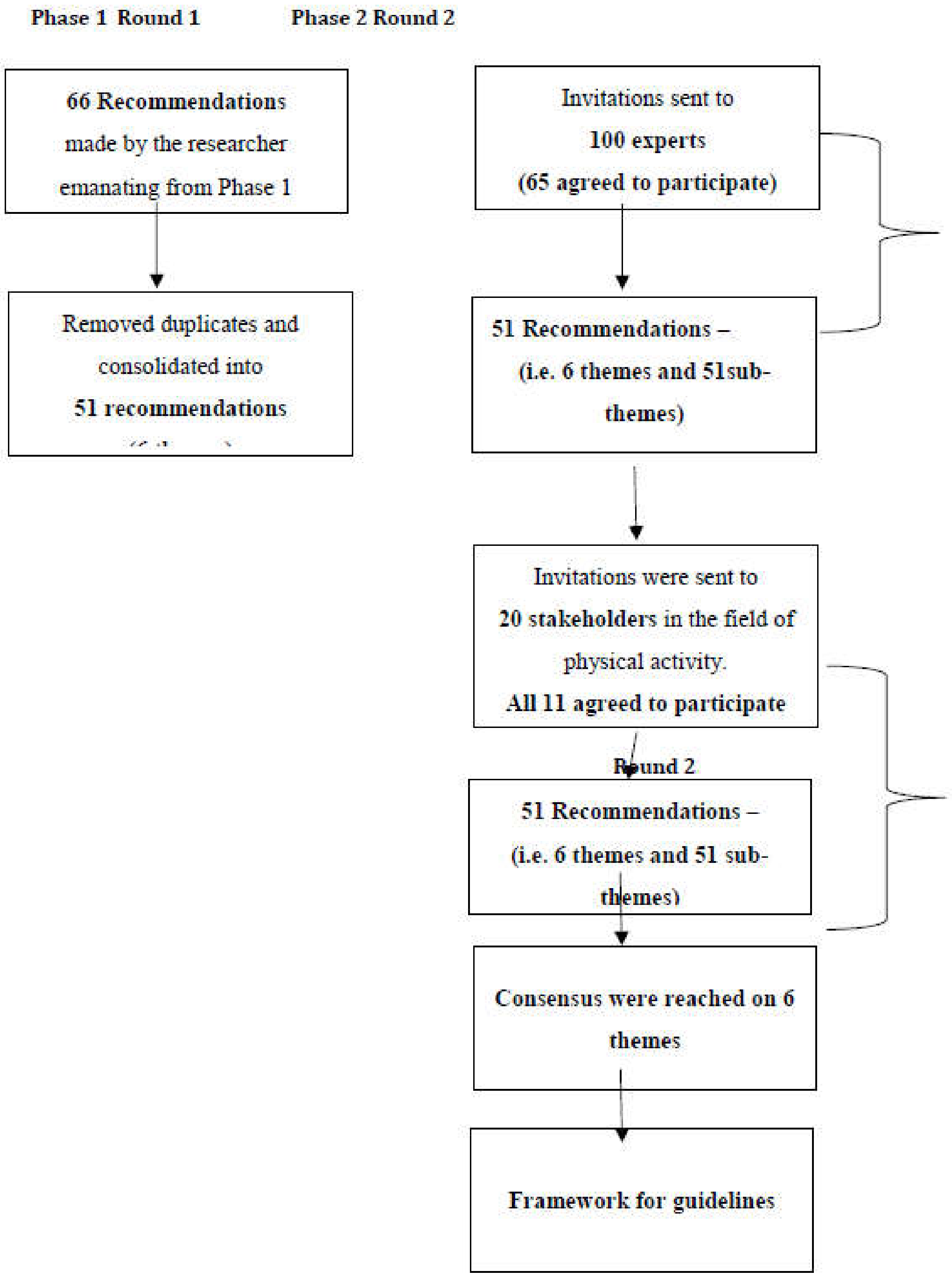

Figure S1. Consensus framework.

Table S1. Framework for guidelines developed for the current study.

Themes and Sub-themes
Theme 1 A: Key guidelines for adolescents
Adolescents should do:<br>60 min of Physical Activity (PA) activity per day for 3-5 days.
Parents should participate for 150-300 minutes for 3-5 days per week.
20-minute bouts of PA have a cumulative effect.
Theme 1 B: Key guidelines for parents
Help adolescents to set realistic PA goals.
Make use of a variety of PA options.
Free-play remains a popular option.
Parents to create opportunities for social PA settings.
Get involved and set realistic goals.
Theme 3: Resource for parents: Physical activity preferences
Type, examples, duration intensity, level of PA (Activity-Table included below)
Theme 4 A: Parental involvement Guidelines
Be actively involved in school sport.
Be involved with planning for PA and preparation before events.
Go and watch them participate (spectator parents).
Theme 4 B: Parental involvement Guidelines
Show an interest in what adolescents do.
Respect adolescent's choices PA.
Participate in PA with your adolescent.
Allow adolescents to choose/ select their own activities.
Parents support adolescents with sports equipment, financial support, transport fees, support with overcoming barriers to participation.
Personal protective gear is something worn by a person to protect a specific body part: (helmets, eyewear, goggles, shin-guards, elbow & knee pads, and mouth- guards masks).
Theme 4 C: Parental involvement Guidelines (Directing behaviour)
Set realistic boundaries for PA participation.
Open communication with adolescents is needed to establish:<br>Ground-rules, Curfews, Boundaries, PA time, Limit screen time/ sedentary time.
Parents listen to your adolescent too they are savvy and knowledgeable.
Theme 4 D: Parental involvement Guidelines (Parental encouragement)
Provide positive feedback and motivate adolescents.
Good role models in parents, caregivers, and teachers should model and encourage an active lifestyle for children.
Praise, reward, and encourage adolescents to be active.
Being active as a family is a great way to model and encourage physical activity.
Theme 4 E: Parental involvement Guidelines (Parental awareness of the benefits)
Improved cognitive function.
Reduced risk of cancer.
Brain health benefits and improved cognitive function.
Reduced anxiety and depression risk.
Improved sleep and quality of life.
Both aerobic and muscle-strengthening physical activity are beneficial.
The health benefits for people with chronic and health conditions.
Endorphins/ feel good/ self-concept/ self-image
Theme 5 A: Safe PA in stressed environments
PA risks must be understood by parents.

Select types of PA that are appropriate for level of fitness.
Screen the PA environments for safety risks. know what they want to doing.
Consult a health care professional before starting with PA if adolescents have health conditions (types and amounts of PA).
Theme 5 B: Safe PA in stressed environments (Personal safety)
Parents assisting adolescents to sensible choices (when, where, how to be active).
Teach adolescents to be aware of their surroundings and to be alert and avoid risky situations.
Establish a buddy system for PA.
Join a walking bus to and from school.
Theme 5 C: Safe PA in stressed environments (Personal safety)
Physical separation from motor vehicles and awareness of surroundings.
Choose places that are well lit.
Following rules and safety rules is the best way to reduce activity-related injuries.
Theme 6 A: Sustained PA (Taking-Action)
Provide time for both structured and unstructured PA
PA through break time/ recess.
The use of technology and digital tools to use during PA sessions is preferred by adolescents.
Online activities yoga, games, programs would interest adolescents
Theme 6 B: Sustained PA guidelines
Start gradually and be consistent.
Start PA at a young age and make it a lifestyle.
Everyone has a role to play:<br>Schools, Communities, Parent-teacher associations Faith groups, Health groups<br>Businesses, Civic organizations<br>Public safety agencies, Policy makers

Table S2. Framework with particpants comments.

Draft guidelines			
No.	Item	Consensus and Response rate	Workshop feedback
Theme 1: Key guidelines for adolescents			
1	Adolescents should do:<br>60 min of Physical Activity (PA) activity per day for 3-5 days.	90.9%	Consensus
2	Parents should participate for 150-300 minutes for 3-5 days per week.	72.7%	Consensus, participant Murphen suggested that of recommending parental active time suggest that parents facilitate or participate with adolescents in this time
3	20-minute bouts of PA have a cumulative effect.	72.7%	Consensus, Participant 3: Ryan Provide further clarity w.r.t this specific guideline.
Theme 2: Guidelines for parents			
4	Help adolescents to set realistic PA goals.	90.9%	Consensus
7	Make use of a variety of PA options.	72.7%	Consensus

8	Free-play remains a popular option.	90.9%	Consensus
9	Parents to create opportunities for social PA settings.	81.8%	Consensus
10	Get involved and set realistic goals.	18.1%	Consensus
Theme 3: Resource for parents: Physical activity preferences			
1	Type, examples, duration intensity, level of PA (Activity-Table included below)	100%	Table related to types, duration was well received. Participant Clyde/ Ryan suggested that the time be allocated in the table.
Theme 4: Parental involvement Guidelines			
1	Be actively involved in school sport.	81.8	Consensus
2	Be involved with planning for PA and preparation before events.	90.9%	Consensus
3	Go and watch them participate (spectator parents).	100%	Consensus
Theme 4: Parental involvement Guidelines (parental support)			
4	Show an interest in what adolescents do.	100%	Consensus
5	Respect adolescent's choices PA.	90.9%	Consensus
6	Participate in PA with your adolescent.	81.8%	Consensus
7	Allow adolescents to choose/ select their own activities.	72.7%	Consensus
8	Parents support adolescents with sports equip-ment, financial support, transport fees, support with overcoming barriers to participation.	81.8%	Consensus
9	Personal protective gear is something worn by a person to protect a specific body part: (hel-mets, eyewear, goggles, shin-guards, elbow & knee pads, and mouth- guards masks).	100%	Consensus
Theme 4: Parental involvement Guidelines (Directing behaviour)			
10	Set realistic boundaries for PA participation.	100%	Consensus
11	Open communication with adolescents is needed to establish:<br>Ground-rules.<br>Curfews.<br>Boundaries.<br>PA time.<br>Limit screen time/ sedentary time.	100%	Consensus
12	Parents listen to your adolescent too they are savvy and knowledgeable.	100%	Consensus
Theme 4: Parental involvement Guidelines (Parental encouragement)			
13	Provide positive feedback and motivate adolescents.	81.8%	Consensus
14	Good role models in parents, caregivers, and	90.9	Consensus

	teachers should model and encourage an active lifestyle for children.		
15	Praise, reward, and encourage adolescents to be active.	100%	Consensus
16	Being active as a family is a great way to model and encourage physical activity.	100%	Consensus
Theme 4: Parental involvement Guidelines (Parental awareness of the benefits)			
17	Improved cognitive function.	100%	Consensus, Participants agree that benefits need to be included and that there is a lack of awareness of the basic benefits of PA.<br>Dawn, Janice<br><br>More benefits exist and need to be included
18	Reduced risk of cancer.		
	Brain health benefits and improved cognitive function.		
	Reduced anxiety and depression risk.		
	Improved sleep and quality of life.		
	Both aerobic and muscle-strengthening physical activity are beneficial.		
	The health benefits for people with chronic and health conditions.		
	endorphins/ feel good/ self-concept/ self-image		
Theme 5: Safe PA in stressed environments			
1	PA risks must be understood by parents.	100%	Consensus
2	Select types of PA that are appropriate for level of fitness.	100%	
3	Screen the PA environments for safety risks. know what they want to doing.	72.7%	
4	Consult a health care professional before starting with PA if adolescents have health conditions (types and amounts of PA).	100%	consensus
Theme 5: Safe PA in stressed environments (Personal safety)			
5	Parents assisting adolescents to sensible choices (when, where, how to be active).	100%	
6	Teach adolescents to be aware of their surroundings and to be alert and avoid risky situations.	100%	Consensus on both
7	Establish a buddy system for PA.	100%	
8	Join a walking bus to and from school.	100%	consensus
Theme 5: Safe PA in stressed environments (Community Safety)			
9	Physical separation from motor vehicles and awareness of surroundings.	100%	
10	Choose places that are well lit.	80.8%	
11	Following rules and safety rules are the best way to reduce activity-related injuries.	90.9%	
Theme 6: Sustained PA (Taking-Action)			
12	Provide time for both structured and unstructured PA	100%	62. Explain more here says

13	PA through break time/ recess.	90.9%	Clyde<br>64. Unclear, re-write	
14	The use of technology and digital tools to use during PA sessions is preferred by adolescents.	100%		
15	Online activities yoga, games, programs would interest adolescents	100%	Include add	Comment made by Mickel
Sustained PA guidelines				
16	Start gradually and be consistent.	100%	Consensus	
17	Start PA at a young age and make it a lifestyle.	100%	Consensus	
18	Everyone has a role to play:<br>Schools<br>Communities<br>Faith groups<br>Businesses<br>Civic organizations<br>Parent-teacher associations<br>Health groups and<br>Public safety agencies<br>Policy makers	90.9%	Consensus	

Table S3. Final Resource table A6: The approved consensus table clarifies basic activities and provides parents with an easy to follow template of possible activities to do with adolescents.

**Type of activity including definition and example**	**Frequency (how often)**	**Duration (how long to do the activity)**	**Intensity (how hard to work)**
**Aerobic activities defined**: activities that make the heart beat faster.<br>**example**: run, brisk-walk, walk, swim, hiking, dance, free-play, skipping, gymnastics	3 days<br>Per week	60 minutes per session, or, 20 minute-bouts of activity repeated 3 times.	Moderate or vigorous activity depending on your fitness level. Start slow and build up gradually.
**Muscle strengthening** activities defined: activities linked to strengthening the muscles.<br>**examples**: climbing activities making use of one's own<br>body weight.	2 days<br>Per week	20 minute-bouts of activity repeated 3 times. Start slow and build up gradually.	Moderate or vigorous activity depending on your fitness level. Start slow and build up gradually.
**Bone strength activities** defined as: activities selected by adolescents in the findings of the study and categorized here as bone strength.<br>**examples:** jumping jacks, running, brisk walking,	2 days<br>Per week	20 minute-bouts of activity repeated 3 times. Start slow and build up gradually.	Moderate or vigorous activity

activities using one's own body weight, pushing and lifting activities, moderate and vigorous house-work, Tennis, hopscotch and free-play.			
**Balance and flexibility** activities: activities preferred by adolescents in the current study and categorized here as balance and flexibility activities. **examples:** movement and rhythmic movement activities, dance, gymnastics, whole-body stretching, walk the line, walk backwards in free-play, balancing on one leg, proprioception, balancing on foam.	Do these activities daily	bouts of activity, start slow and build up gradually.	Moderate or vigorous activity
Warm up and cool down defined as light activities used to prepare the body for activity session. It can take the form of active warm up or passive warm up activities. Example: walking on the sport, cycling slow and gradually warming the body up. The aim is that the heart rate must increase gradually. Cooling down help to lower the heart rate. Cooling down and stretching combine help alleviate muscle aches and pains.	Daily before and after every session	5-15 minutes before and after every session.	Light
